# Patterns of methylation and transcriptional plasticity during thermal acclimation in a reef‐building coral

**DOI:** 10.1111/eva.13757

**Published:** 2024-07-17

**Authors:** Leslie Guerrero, Rachael Bay

**Affiliations:** ^1^ University of California, Davis Davis California USA

**Keywords:** acclimation, coral, DNA methylation, gene expression plasticity

## Abstract

Phenotypic plasticity can buffer organisms against short‐term environmental fluctuations. For example, previous exposure to increased temperatures can increase thermal tolerance in many species. Prior studies have found that acclimation to higher temperature can influence the magnitude of transcriptional response to subsequent acute thermal stress (hereafter, “transcriptional response modulation”). However, mechanisms mediating this gene expression response and, ultimately, phenotypic plasticity remain largely unknown. Epigenetic modifications are good candidates for modulating transcriptional response, as they broadly correlate with gene expression. Here, we investigate changes in DNA methylation as a possible mechanism controlling shifts in gene expression plasticity and thermal acclimation in the reef‐building coral *Acropora nana*. We find that gene expression response to acute stress is altered in corals acclimated to different temperatures, with many genes exhibiting a dampened response to heat stress in corals pre‐conditioned to higher temperatures. At the same time, we observe shifts in methylation during both acclimation (11 days) and acute heat stress (24 h). We observed that the acute heat stress results in shifts in gene‐level methylation and elicits an acute transcriptional response in distinct gene sets. Further, acclimation‐induced shifts in gene expression plasticity and differential methylation also largely occur in separate sets of genes. Counter to our initial hypothesis no overall correlation between the magnitude of differential methylation and the change in gene expression plasticity. We do find a small but statistically significant overlap in genes exhibiting both dampened expression response and shifts in methylation (14 genes), which could be candidates for further inquiry. Overall, our results suggest transcriptional response modulation occurs independently from methylation changes induced by thermal acclimation.

## 1 INTRODUCTION

Increased intensity and frequency of climate anomalies has led to physiological stress, population decline, and species redistribution across the globe. One potential mechanism for buffering against extreme climate fluctuations is adaptive phenotypic plasticity–phenotypic changes occurring within an individual's lifetime that promote greater fitness in response to environmental triggers (Gienapp et al., 2008; Merilä & Hendry, 2014). The ability of organisms to undergo phenotypic plasticity is seemingly ubiquitous across the tree of life (Agrawal, 2001; Gotthard & Nylin, 1995; Santillán & Mackey, 2008; Sorek & Cossart, 2010; Sultan, 2000), and plastic phenotypes can vary widely among taxa, including shifts in physiology, morphology, or behavior (Ghalambor et al., 2010; Sultan, 2000). The degree of plasticity can vary between individuals, populations, and species demonstrating the diversity and evolvability of trait plasticity (Dingemanse & Wolf, 2013; Gunderson & Stillman, 2015; Kelly, 2019; Kenkel & Matz, 2016; Mallard et al., 2020; Putnam et al., 2016). Climate change is predicted to interfere with the reliability of environmental stimuli for plastic responses in natural populations by increasing climate variability or forcing species redistributions, shifting selection pressure on phenotypic plasticity (Bonamour et al., 2019; Kelly, 2019). Accordingly, building a mechanistic understanding of adaptive plasticity can aid in predictions of when adaptive plasticity might be sufficient to buffer against the ongoing effects of climate change.

Phenotypic plasticity often results from gene expression shifts caused by environmental triggers (Schlichting & Pigliucci, 1993, 1995; Schlichting & Smith, 2002). Although differing baseline gene expression is associated with variation in several climate‐relevant phenotypes (Dayan et al., 2015; DeBiasse & Kelly, 2016; Gibbons et al., 2017; Hamdoun et al., 2003), recent evidence from transcriptomic studies reveals that the magnitude of gene expression response to climatic stressors, rather than the constitutive level of expression, may determine the outcome. For example, four genotypes of wheat seedlings acclimated to drought had a reduced physiological response to a 48‐h water stress assay and lower magnitudes of expression of drought response genes than the non‐acclimated counterparts (Amoah et al., 2019). Similarly, plasticity in thermal tolerance (i.e., thermal acclimation) in corals followed by short‐term thermal exposure was associated with a reduced gene expression response of heat‐stress genes (Bay & Palumbi, 2015; Bellantuono et al., 2012). Lastly, a reciprocal transplant experiment revealed that a higher capacity for transcriptional plasticity in a coral population was associated with survival during thermal stress (Kenkel & Matz, 2016). Together these studies highlight the potential for gene expression plasticity to facilitate adaptive phenotypic plasticity. However, mechanisms driving such transcriptional modifications remain elusive (Barshis et al., 2013; Gleason & Burton, 2015; Hamdoun et al., 2003; Logan & Cox, 2020; López‐Maury et al., 2008; Mallard et al., 2020). How environmental signals are translated to changes in gene expression plasticity remains a fundamental question. Understanding these mechanisms will aid in predicting when we expect adaptive plasticity to occur and the potential limits.

DNA methylation offers a potential intermediate between environmental change and gene expression plasticity (Eirin‐Lopez & Putnam, 2019). DNA methylation—a stable yet reversible covalently attached methyl group to nucleotides—is an epigenetic mechanism that putatively affects gene expression plasticity by interacting with transcriptional regulators (Jones, 2012; LaSalle et al., 2013). DNA methylation dynamics are influenced by the environment and these shifts are associated with a genotype's potential for phenotypic plasticity (Dixon et al., 2018; Putnam et al., 2016). Interestingly, methylation distribution and density vary among genomic features within a single genome and are associated with different gene expression patterns. For instance, the methylation of cytosines in cytosine‐guanine dinucleotide motifs (CpG) within coding sequences, or gene body methylation, is associated with hypomethylated promoters and correlated with expression magnitude and variation in invertebrate species (Dixon et al., 2018; Dixon & Matz, 2022; Jones, 2012; Li et al., 2018). In particular, genes regularly expressed to maintain basal homeostatic processes or housekeeping genes tend to be more highly methylated than environmentally responsive genes (Dixon et al., 2018; Gatzmann et al., 2018). In cnidarians, heavily methylated intragenic transposable elements can explain hypermethylated gene bodies (Ying et al., 2022). However, high methylation of intragenic transposable elements in cnidarians does not affect gene expression levels (Ying et al., 2022). Altogether these studies highlight the complex relationship between DNA methylation and gene expression patterns.

Despite links between baseline methylation and gene expression (Anastasiadi et al., 2018; Dixon & Matz, 2022; Gatzmann et al., 2018), whether environmentally‐induced shifts in methylation lead to shifts in gene expression is unclear. By coupling transcriptome and methylome analyses across six invertebrate species, Dixon and Matz (2022) show that changes in gene body methylation do not explain global changes in gene expression. A common‐garden experiment performed on full‐sibling families of *Crassostrea virginica* show that, while gene expression patterns are driven by sampling location, DNA methylation is driven by genetic differences between families, suggesting methylation does not facilitate global changes in gene expression (Johnson et al., 2021). Comparative analysis between *Arabidopsis thaliana* and *Eutrema salsugineum*, a species that has lost gene body methylation, shows gene function and histone modifications are indistinguishable between the two species suggesting a minimal relationship between gene body methylation and transcription (Bewick et al., 2016; Muyle & Gaut, 2019; Muyle et al., 2022). Nevertheless, gene body methylation is conserved across plants, fungi, and animals, suggesting functional significance despite conflicting conclusions about the relationship with gene expression (Dixon & Matz, 2022; Entrambasaguas et al., 2021; He et al., 2020; Muyle et al., 2021; Zeng et al., 2018).

Here, we examine the association between gene body methylation and transcriptional plasticity observed during rapid thermal acclimation in the reef‐building coral species *Acropora nana*. Particularly vulnerable to ocean warming, reef‐building coral species within the Acropora genus are of significant conservation concern due to bleaching‐induced mortality following acute warming events. As long‐lived benthic marine invertebrates, thermal acclimation, the ability to increase thermal tolerance following exposure to sublethal elevated temperatures, is one of the few mechanisms corals have to respond to short‐term environmental fluctuations (Middlebrook et al., 2008). Thermal acclimation is associated with altered magnitudes of transcriptional response to subsequent acute heat stress, a phenomenon we refer to as “transcriptional response modulation”. This modulation in coral heat response genes during imminent acute thermal challenge suggests the presence of molecular pathway(s) that presumably (i) preserve the memory of previous thermal exposure and (ii) mediate an altered transcriptional response leading to stress resilience (Bay & Palumbi, 2015; Bellantuono et al., 2012; Hackerott et al., 2021). We leveraged a previously published experiment in which *Acropora nana* fragments were acclimated to elevated but sublethal temperatures and then treated with an acute heat stress assay (Bay & Palumbi, 2015). Bleaching under acute heat stress was reduced in acclimated individuals (Bay & Palumbi, 2015). Bay and Palumbi then measured gene expression following the heat stress assay to characterize the transcriptional response to heat stress between acclimated and non‐acclimated individuals. Within that experiment, individuals acclimated to higher temperatures were shown to have a reduced transcriptional response to heat stress. For the subset of the genes exhibiting a modulated expression response to heat stress, we aimed to explore whether these genes undergo changes in methylation during the acclimation treatment leading up the heat stress assay. Simultaneously investigating gene body methylation and gene expression patterns associated with increased thermal tolerance following acclimation, we addressed the following questions: (i) Are there shifts in gene body methylation during acclimation to elevated temperatures? (ii) Do shifts in methylation correspond to reduced transcriptional plasticity in the same genes?

## 2 METHODS

### 2.1 Experimental design

**FIGURE 1:**
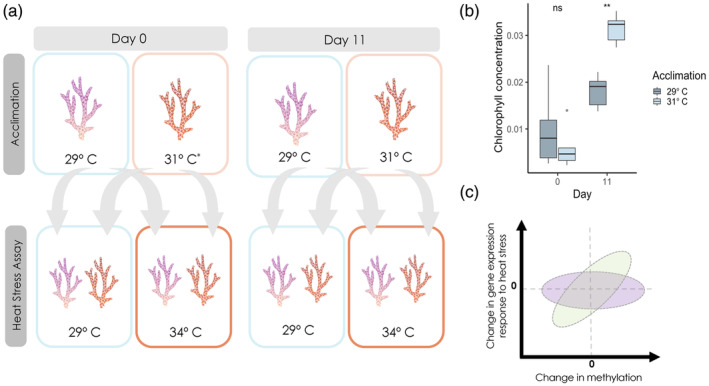
(a) Experimental design showing the acclimation treatments and how samples from each acclimation treatment were distributed for the heat stress assay. *Day 0 samples in the 31°C tank never actually experienced 31°C acclimation. (b) Plot of *chlorophyll a* concentration redrawn from previously performed study. Samples in the 31°C (Day 11) acclimation tank have a higher *chlorophyll a* concentration following the 5‐h 34°C heat stress assay. (c) Prediction of the relationship between change in gene expression response to heat stress following acclimation and the change in average percent DNA methylation. The green ellipse symbolizes the hypothesized positive association between these variables. The purple ellipse symbolizes the alternative hypothesis if there is no relationship between the variables.

The samples used in this study were taken from a previously published acclimation experiment, and details on the experimental setup can be found in that paper (Figure 1a; Bay & Palumbi, 2015). Briefly, we took whole small colonies of *Acropora nana* (Studer, 1878) from the reef on Ofu Island, American Samoa, and placed them in outdoor aquaria for acclimation. Three colonies were placed in each tank, with two tanks per acclimation treatment, totaling six colonies per acclimation treatment and 18 colonies in total. Due to logistical constraints, genotype was not replicated across acclimation treatments. The original study had an ambient (29°C), elevated (31°C), and variable (29–33°C daily) acclimation treatment to mimic in situ thermal fluctuation. We only used samples from the two stable treatments (29°C and 31°C) for our purposes. This 2°C temperature difference was sufficient to induce thermal acclimation and is within the typical thermal range. At different time points throughout the experiment, we sampled branches from each colony and subjected them to an acute heat stress assay to test thermal tolerance. For the heat stress assay, two branches were sampled from each colony; one was held at an ambient control temperature (29°C) for 24 h, whereas the other was subjected to heat stress consisting of a 3‐h ramp to 34°C followed by 5 h at 34°C, then a decline to 29°C for approximately 1 h. This temperature profile was designed to mimic tidal fluctuations in temperature at this location and expose variation in bleaching among samples. The heated samples were incubated at 29°C overnight for the remainder of the 24‐h assay. At 6:00 AM the following morning, branches were cut in half and preserved in 95% ethanol and RNA stabilizing solution (70 g ammonium sulfate/100 mL solution, 10 mM EDTA, 25 mM sodium citrate, 5.4 pH) for downstream analysis. To assess thermal tolerance after heat stress, we measured chlorophyll concentration as a proxy for bleaching (Ritchie, 2008). Figure 1b, redrawn from Bay and Palumbi (2015), shows the increase in thermal tolerance (reflected in higher chlorophyll content of heat‐stressed samples) for 31°C acclimated corals than 29°C acclimated corals after 11 days. In this study, we examine samples in the acclimation treatments for either 0 days (i.e., collected the previous day and held at 29°C overnight) or 11 days. Note that this means the Day 0 samples in the 31°C tank never actually experienced 31°C acclimation.

### 2.2 RNASeq

Six samples per treatment group, totaling 48 samples, were extracted and sequenced for RNASeq. Sample preservation, library prep, cDNA sequencing, and read trimming are described in Bay and Palumbi (2015). We aligned trimmed reads to the *Acropora millepora* genome using STAR aligner software (v. 2.7.0e) (Dobin et al., 2013; Fuller et al., 2020). Filtering parameters that optimized alignment were: ‐‐outFilterScoreMinOverLread 0, ‐‐outFilterMatchNminOverLread 0, ‐‐outFilterMatchNmin 0, ‐‐outFilterMismatchNmax 4. We used HTSeq v. 0.9.1 to count all reads that mapped to genes, including reads that map to multiple genes(‐‐nonunique all), using the annotated gene models provided with the *Acropora millepora* reference genome (Anders et al., 2015; Fuller et al., 2020). We expect that a congener alignment may have resulted in some reads failing to map due to the divergence between *Acropora millepora* and *Acropora nana*, thus, these reads were excluded from the remainder of the analyses.

To quantify the symbiont composition within each sample, we aligned RNASeq reads to Symbiodinium goreaui (GenBank accession number: AF333515) and Durusdinium trenchii (GenBank accession number: LC718590) ITS2 sequences using the STAR aligner software (v. 2.7.0e) (Dobin et al., 2013; LaJeunesse, 2001; Mihirogi et al., 2023). We used the STAR option ‐‐outFilterMultimapNmax 1 to ensure that STAR reports only the best alignment for each read. We counted the number of reads that uniquely aligned to each ITS2 sequence in each sample.

### 2.3 Whole‐genome bisulfite sequencing

We extracted DNA from the same branches used for RNA‐Seq (six samples per treatment group, totaling 48 samples) using the Qiagen DNeasy Blood and Tissue Kit (Cat. No. 69504). Genomic DNA was sent to Novogene (Sacramento), and Methyl‐MaxiSeq libraries were prepared from 300 ng of genomic DNA digested with 2 units of Zymo Research's dsDNA Shearase™ Plus (Cat. No. E2018‐50). The fragments produced were end‐blunted, 3′‐terminal‐A extended, then purified using the Zymo Research DNA Clean & Concentrator™ kit (Cat. No. D4003). The A‐tailed fragments were ligated to pre‐annealed adapters containing 5′‐methylcytosine instead of cytosine. Bisulfite treatment of the fragments was done using the EZ DNA Methylation–Lightning kit (Zymo Research, Cat. No. D5030). PCR was performed with Illumina TruSeq indices, and the size and concentration of the fragments were confirmed on the Agilent 2200 TapeStation. Before sequencing, samples were spiked with Illumina's PhiX Control library. Sequencing was performed using the Novaseq 6000 platform with Paired‐End 150 (PE150) reads, aiming for a target coverage of 30× based on the 500 Mb *Acropora* millepora estimated genome size. The average raw read coverage achieved was 23×.

To analyze WGBS reads, we used Trim Galore! Version 0.6.3 to filter out reads that were <20 nt long along with their read‐mate (Krueger, 2023). We clipped the 5′ ends of the reads to remove possible methylation bias (‐‐clip_R1 10 ‐‐clip_R2 10). We aligned trimmed reads to the *Acropora millepora* genome using bwa‐meth with default settings (Fuller et al., 2020; Pedersen et al., 2014). We used SAMtools view (Version 1.9) to filter by excluding unmapped reads, mapped reads with unmapped mates, and/or reads that failed platform quality thresholds (−F 524) (Li et al., 2009). We kept reads with a minimum mapping quality of 2 (−q 2) (Li et al., 2009). To filter out PCR duplicates, we used Picard MarkDuplicates (Version 2.20.2) with default settings (Picard, 2023). Finally, to extract methylation calls, we used MethylDackel (https://github.com/dpryan79/MethylDackel). This output summarizes the strand‐specific frequency of cytosines and thymines (a proxy for unmethylated cytosine). In downstream statistical analyses, these frequencies are converted to a percentage of DNA methylation at each CpG dinucleotide. As we did not have full genomic data for *A. nana*, we were unable to mask possible C to T substitutions between the *A. millepora* reference genome and *A. nana* samples. The minimum depth of methylation calls was set to 10, and the maximum variant fraction was set to 0.75 to exclude possible non‐cytosine alleles at reference CpG sites.

### 2.4 Differential gene expression and heat response gene identification

We analyzed differential gene expression using R Package DESeq2 v. 1.36.0 (Love et al., 2014; R Core Team, 2022). First, we filtered for genes with a depth of at least 10 reads in all samples and normalized data with the variance stabilizing transformation. We performed a PCA to investigate the relationship between acclimation and heat stress treatment groups. We identified heat response genes by comparing the heat‐stressed samples to their corresponding control counterparts that had not undergone thermal acclimation (29°C at Day 0 and Day 11, and 31°C at Day 0). Since thermal acclimation can lead to the downregulation of a subset of stress response genes, transcriptional data from thermally acclimated samples were excluded from identifying the heat response genes. We calculated the log_2_ fold‐change between heat‐stressed and control samples and performed log_2_ fold‐change shrinkage with the ‘ashr’ method to account for the strong variation in log_2_ fold‐change associated regions of low read counts (Love et al., 2014; Stephens, 2017). Heat response genes are defined by a |log_2_ fold‐change| >2 and Benjamini‐Hochberg false discovery rate (BH *p*_adj_) < 0.01. We classified a total set of heat response genes from the union of the significant differential expression within any of the three sample sets: 29°C (Day 0 and Day 11) and 31°C (Day 0). A more conservative set of “core” heat stress genes were identified by the intersection of the significant heat stress genes across all three contrasts. We analyzed Gene Ontology (GO) terms with the R package, topGO v. 2.50.0 (Alexa & Rahnenfuhrer, 2023). We compared the list of “core” heat response genes against a background of all genes that passed our quality filters. Enriched GO terms were identified with the classic Fisher's test with a *p* <0.01 and at least 10 transcripts within each category.

### 2.5 Quantifying transcriptional response modulation

Previously, we found no change in gene expression during acclimation but rather a change in the magnitude of gene expression response to heat stress in corals pre‐conditioned at higher temperatures (Bay & Palumbi, 2015). Here, we quantified this “transcriptional response modulation” using R Package DESeq2 v. 1.36.0 (Love et al., 2014; R Core Team, 2022). DESeq2 estimates the interaction term coefficient for each gene and uses the Wald test to test if the interaction term is statistically different from zero (Love et al., 2014). The predictors of the coefficient terms are the levels of the experimental design (Love et al., 2014). In our case, the experimental conditions are acclimation temperature and heat stress treatment, with the Wald test quantifying the interaction between the two. To avoid the confounding factor of Day, we used heat stress treatment and control samples from the acclimation temperature control, 29°C (Day 11), and the acclimation treatment, 31°C (Day 11), for comparison to capture the effect of thermal acclimation. We identified two categories of transcriptional response modulation: amplified and dampened expression. Transcripts with amplified expression had a higher magnitude of expression response induced by heat stress in samples acclimated to 31°C than those acclimated to 29°C (Wald statistic >0 and BH *p*_adj_ <0.1). Transcripts with dampened expression had a smaller magnitude of expression response (Wald statistic <0 and BH *p*_adj_ <0.1) (Love et al., 2014). To test if amplified and dampened genes have different mean expressions, we log_10_‐transformed each transcript's average normalized count values and performed a two‐sample *t*‐test. We analyzed Gene Ontology (GO) terms with the R package, topGO v. 2.50.0 (Alexa & Rahnenfuhrer, 2023). We compared lists of amplified and dampened genes against a background of all genes that passed our quality filters. Enriched GO terms were identified with the classic Fisher's test with a *p* <0.01 and at least 10 transcripts within each category.

### 2.6 Effects of treatment on DNA methylation

We used methylKit to analyze CpG methylation calls in R (Akalin et al., 2012; R Core Team, 2022). We maintained CpG sites in the ‘methylRawDB’ if there was a coverage count minimum of at least 10, had a maximum cut‐off in the 99.9 percentile of read counts covering the site, and was covered in all the samples. We performed principal coordinate analysis (PCoA) using the Bray–Curtis dissimilarity of DNA methylation sites to visualize the relationship between acclimation and heat stress treatment groups. We recalculated the PCoA after removing the following four outliers: 29°C acclimation treatment (Day 11) heat stress sample replicate 1, 29°C acclimation treatment (Day 11) heat stress control replicate 1, 31°C acclimation treatment (Day 11), heat stress treatment replicate 1 and 31°C acclimation treatment (Day 11), heat stress treatment replicate 3.

We assessed the distribution of methylation across CpG sites and genes in our data set. We used methylKit to summarize percent methylation across CpG sites and genes (Akalin et al., 2012). CpG sites were included in the ‘methylRawDB’ if there was a coverage count minimum of at least 10 and had a maximum cut‐off in the 99.9 percentile of read counts covering the site. For the CpG site methylation distribution, we used the methylKit ‘unite’ function to collate the methylation data and calculate the methylation percentage where the site was covered in at least three replicates per treatment from distinct colonies. For the gene‐level methylation distribution, we created a GRanges object by importing annotated *A. millepora* gene models furnished with the reference genome (Fuller et al., 2020; Lawrence et al., 2009). Then we used the methylKit ‘unite’ function to collate the methylation data, integrate the methylation data with the GRanges object, and calculate the methylation percentage where the gene was covered in at least 3 replicates per treatment.

Previous studies have found that environmental shifts can alter global methylation levels (Metzger & Schulte, 2017; Putnam et al., 2016). We conducted distinct tests to examine heat stress and acclimation induced effects within our dataset. We combined methylation data across all samples with the methylKit ‘unite’ function (Akalin et al., 2012; Fuller et al., 2020; Lawrence et al., 2009). We calculated the average percent of methylation at each CpG site covered in at least three replicates per treatment across all treatment groups. We excluded missing data from the average methylation percent calculation. We tested the effect of heat stress and acclimation treatments on global CpG methylation percent with an ANOVA. An important caveat is that since we were unable to account for C‐to‐T substitutions in our data, these substitutions may artificially increase unmethylated calls at CpG sites, potentially resulting in decreased variance in methylation (Methods in Data S1). Although this artifact could affect baseline methylation levels, it should not impact inferences about differential methylation, though artificial reduction in methylation variance due to C–T polymorphisms could also result in false positives.

We tested whether DNA methylation percent varies between genomic features and if there were feature‐specific changes in methylation following acclimation. First, we were interested in the methylation percentage within and between genomic features. We created a GRanges object by importing annotated *A. millepora* exons and introns (Fuller et al., 2020; Lawrence et al., 2009). We created a GRanges object by importing BED files that define predicted coordinates of promoters, transcription start sites (TSS), long interspersed nuclear element (LINE) repeats, short interspersed nuclear element (SINE) repeats, and rolling circle (RC) repeats in the *A. millepora* genome (https://github.com/Groves‐Dixon‐Matz‐laboratory/benchmarking_coral_methylation/tree/master/windowStats) (Dixon & Matz, 2021). We integrated the feature‐specific GRanges objects with methylation data across all samples and calculated the methylation percentage where the region was covered in at least three replicates per treatment with the methylKit ‘unite’ function (Akalin et al., 2012). We calculated the average percent of methylation at each promoter, TSS, exon, intron, LINE repeat, SINE repeat, and RC repeat across all samples. We excluded missing data from the average methylation percent calculation. We used an ANOVA followed by a Tukey's HSD test to test whether DNA methylation varies across genomic features.

Next, we investigated gene‐level differential methylation due to heat stress. We created a GRanges object by importing annotated *A. millepora* genes and then integrated this object with methylation data across all samples with the methylKit ‘unite’ function (Akalin et al., 2012; Fuller et al., 2020; Lawrence et al., 2009). We set ‘min.per.group’ to 3 and performed gene‐level differential methylation analysis using methylKit's ‘calculateDiffMeth’ function to determine genes with different methylation between heat‐stressed and control groups (Akalin et al., 2012). The heat assay and control groups were formed by pooling all heat‐stress and control samples, respectively, across all acclimation treatment groups: 29°C (Day 0), 31°C (Day 0), 29°C (Day 11), and 31°C (Day 11). We set the minimum percentage change threshold to 25% difference between heat‐stressed and control groups. The results were corrected for false discovery rate using a *q*‐value threshold of 0.05. After calculating the change in DNA methylation between heat stress treatments, we analyzed Gene Ontology (GO) terms with the R package, topGO v. 2.50.0 (Alexa & Rahnenfuhrer, 2023). We compared differentially methylated genes against a background of all genes that passed our quality filters. Enriched GO terms were identified with the classic Fisher's test with a *p* <0.01 and at least 10 genes within each category.

We hypothesize that thermal acclimation may lead to changes in DNA methylation, which forms the primary focus of our study. We investigated differences in methylation due to acclimation at the CpG site and gene levels. To capture the effect of thermal acclimation, we formed the acclimation control group by pooling 29°C (Day 11) heat stress treatment and control samples, and we formed the acclimation treatment group by pooling 31°C (Day 11) heat stress treatment and control samples. We used the same filtering criteria as when assaying differences in methylation due to heat stress. After calculating the change in DNA methylation between acclimation and controls, we analyzed GO terms as explained above.

### 2.7 Relationship between gene expression and DNA methylation

First, we tested the relationship between gene expression and average methylation percent within each coding region by performing a linear regression between the log_10_ transformed means of expression counts and the average percent methylation of genes in all samples. To test this relationship, we used the linear regression function in base R where the base mean of expression for each gene was the response variable and the average percent of gene‐level methylation was the predictor variable (R Core Team, 2022). We used ANOVA to determine effect strength and significance.

Then, we tested the relationship between heat stress gene expression plasticity and average methylation percent within each coding region. We used R Package DESeq2 v. 1.36.0 to calculate the log_2_ fold‐change between all heat‐stressed and control samples. All heat‐stressed and control samples were collated with gene‐level methylation percent data. We then performed a linear regression between the gene‐level log_2_ fold‐change standard errors and percent methylation. We used the linear regression function in base R (R Core Team, 2022). The standard error was the dependent variable, and the percent methylation was the independent variable. We used ANOVA to determine effect strength and significance.

### 2.8 Relationship between transcriptional response modulation and change in gene level methylation

Our central hypothesis was that shifts in DNA methylation during acclimation result in transcriptional response modulation. In other words, genes with differential methylation between acclimation treatments would be those whose expression response to acute heat stress was either dampened or amplified in 31°C acclimated samples compared to those acclimated at 29°C. We merged acclimation‐induced differential methylation data with the analysis of transcriptional response modulation (see above). We collated the measure of transcriptional response modulation—the interaction term coefficient—and difference in methylation percent for each gene. We tested the relationship between transcriptional response modulation and change in the gene‐level percent DNA methylation using the linear regression function in base R, where for each gene, the measure of transcriptional response modulation was the dependent variable and methylation difference was the independent variable (R Core Team, 2022). We used ANOVA to determine effect strength and significance. Finally, we tested the difference in average percent methylation of amplified and dampened genes using a two‐sample unpaired Wilcoxon test.

## 3 RESULTS

### 3.1 RNASeq

We aligned RNASeq reads to the *Acropora millepora* reference genome (Fuller et al., 2020). An average of 7.9 million reads (about 56%) of reads uniquely mapped to one locus in each sample. Across all samples, 97,272 reads mapped to symbiodinium ITS2 sequences, with the majority of reads mapping to the *Durusdinium trenchii* ITS2 sequence across all samples (Figure S1).

### 3.2 Gene expression

**FIGURE 2:**
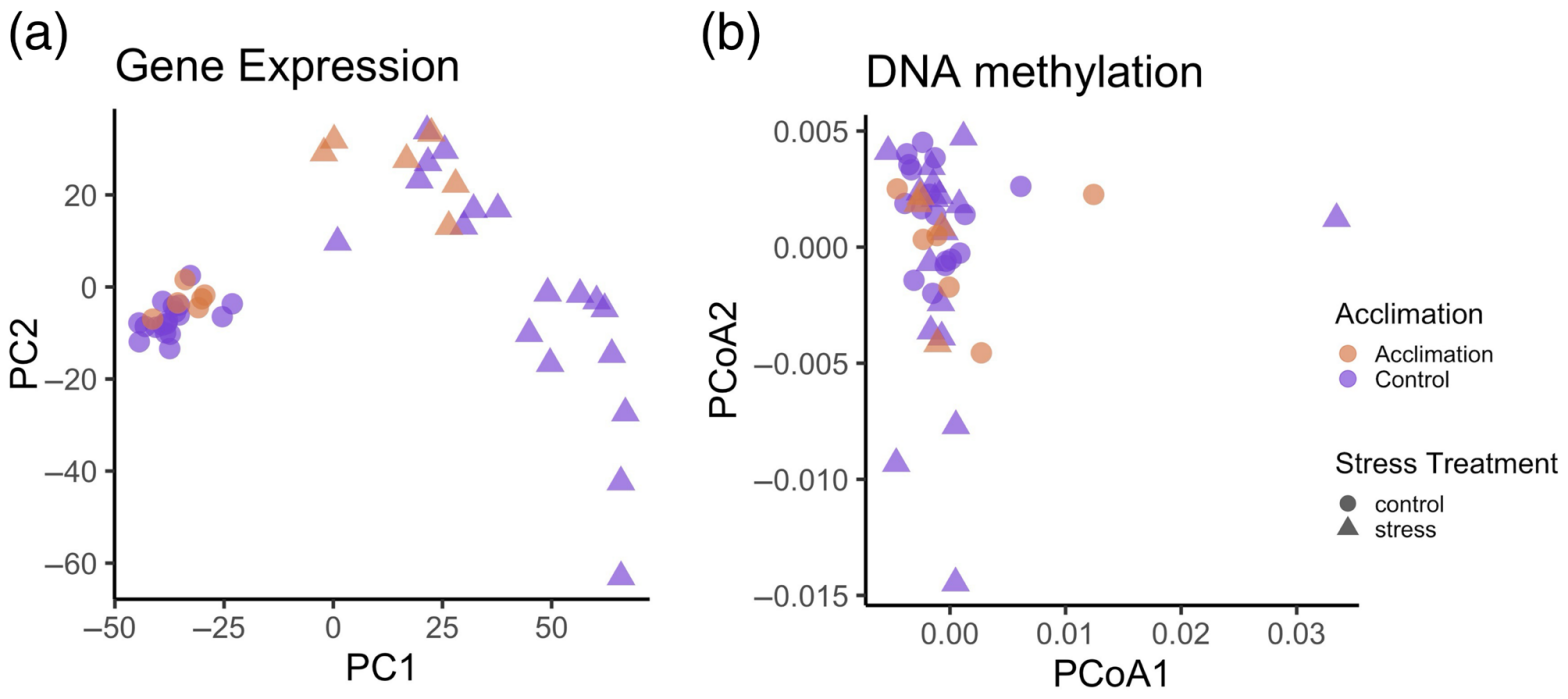
(a) PCA of gene expression showing tight cluster of control samples and variation within heat‐stressed samples along PC2. (b) PCoA of DNA methylation showing no clustering between heat stress and control groups.

We performed differential gene expression analysis to identify heat response genes and to quantify transcriptional response modulation associated with acclimation at higher temperatures. After filtering, 27,501 transcripts were included in the analysis. PCA of gene expression data shows clustering based on heat stress treatment along the first PC axis (Figure 2a). While control samples were tightly clustered, there was more variation within heat‐stressed samples along PC2. While we did not see clustering in the PCA based on acclimation treatment, all 31°C acclimated heat‐stressed samples were among the highest PC2 values, though they overlapped with 29°C acclimated samples. We classified heat response genes by contrasting the control and heat stress sample gene expression. There were 4714 heat response genes from the 29°C (Day 0) samples, 2285 heat response genes from the 31°C (Day 0) samples, and 1368 heat response genes in the 29°C (Day 11) samples. We identified a “core” set of 733 of heat response genes shared across all three contrasts. GO term analysis shows that 98 biological processes GO terms (BP) were enriched in the core set of heat response genes (Table S1). The most significant biological processes were structure and tissue homeostasis and regulation of molecular functions (Table S1). There were also nine molecular function (MF), and six cellular component (CC) GO terms enriched in this set (Table S1).

To identify transcripts with transcriptional response modulation associated with acclimation, we tested for significant interaction between acclimation and acute treatments affecting expression values between heat stress and acclimation treatment. There were 206 amplified genes and 446 dampened genes, supporting our previous finding that response to heat stress was largely dampened in corals acclimated to higher temperatures (Bay & Palumbi, 2015). We found no significant overlap between the core heat response genes and genes with transcriptional response modulation (18 shared genes, chi‐square test, *p* = 0.806). However, we did find a significant overlap between the total set of heat response genes and genes with transcriptional response modulation (299 shared genes, chi‐square test, *p* <7.4e‐51). Biological processes GO term associated with metabolic and transcript processing were enriched in the amplified gene set (Table S2). Cellular component GO terms associated with mitochondrial components and cellular membranes were also enriched in the amplified gene set (Table S2). In the dampened gene set, biological processes GO terms associated with DNA replication and metabolism were enriched, indicating these processes are attenuated following thermal acclimation (Table S3). Molecular functions such as ATP‐dependent activity, DNA binding, and ion transmembrane transporter activity were also reduced following heat stress due to thermal acclimation (Table S3).

### 3.3 Whole‐genome bisulfite sequencing

We performed whole‐genome bisulfite sequencing to assay cytosine‐guanine dinucleotide (CpG) motif methylation across our samples. After filtering for reads that match our threshold for base call and mapping quality, we had an average of 23,342,870 reads per sample. After depth filtering, there were 544,948 methylation calls from a minimum of one sample per treatment group, about 8% of all possible CpG sites. This low percentage is similar to previous reports of genome‐wide DNA methylation in coral and other marine invertebrates (Dixon et al., 2018; Li et al., 2018; Ying et al., 2022). Filtering for sites called in all samples yielded a total of 18,716 CpG sites. We performed PCoA from site calls to assess grouping by treatment. After removing four outliers from a preliminary PCoA (Figure S2), we found no discrete clustering between acclimation or heat assay treatment groups (Figure 2b). Removal of outliers was limited to visualization in Figure 2b, and all samples were included in the remainder of the analyses. Next, we summarized DNA methylation across CpG sites and genes in this dataset. There is a bimodal distribution of the CpG sites, but we do not see a bimodal distribution at the gene level (Figure S3a,b). Previous studies report DNA methylation differences between genomic and intragenic features in corals, so we compared the average DNA methylation across these features in our data (Dixon & Matz, 2021; Liew et al., 2018; Rodriguez‐Casariego et al., 2022; Ying et al., 2022). We find that promoters and exons are hypomethylated relative to introns, LINE repeats, and RC repeats, aligning with previous studies (Figure S3c, Tukey HSD *p* <0.0001) (Liew et al., 2018; Dixon & Matz, 2021; Rodriguez‐Casariego et al., 2022; Ying et al., 2022; but see Li et al., 2018 for DNA methylation of introns and exons in *Exaiptasia pallida*).

We tested global site and gene level changes in percent methylation following heat stress. At the global level, heat stress did not lead to changes in DNA methylation between samples (ANOVA, *F* = 0.7925, *p* = 0.378). However, we did find that heat stress did lead to differential methylation in 451 genes out of a total of 16,531 genes. A total of 205 genes were hypermethylated (≥25% increase), and 246 genes were hypomethylated (≥25% decrease), with significance determined by an FDR‐adjusted *q*‐value of ≤0.05. Five GO terms were enriched in this gene set, and most of these genes code for cellular components associated with intracellular structure and membrane‐bound organelles (Table S4).

We tested global site and gene level changes in percent methylation following acclimation. Acclimation did not lead to global changes in CpG methylation (ANOVA, *F* = 0.6355, *p* = 0.4339). We identified genes with differential methylation between 31°C Day 11 acclimated samples and 29°C Day 11 acclimation control samples. There were 416 differentially methylated genes out of the 15,837 genes where methylation could be summarized in at least three samples per treatment group. A total of 205 genes were hypermethylated, whereas 211 genes were hypomethylated. Two GO terms were enriched in this set of genes: GTPase activity (MF) and GTP binding (MF). GTPases are a protein superfamily associated with many essential cellular pathways in eukaryotes. Finally, there was significant overlap with genes that are differentially methylated following acclimation and heat stress (40 shared genes, chi‐square test, *p* = 4.02e‐12), indicating that thermal treatments largely lead to unique methylation signatures.

### 3.4 Relationship between gene expression and DNA methylation

**FIGURE 3:**
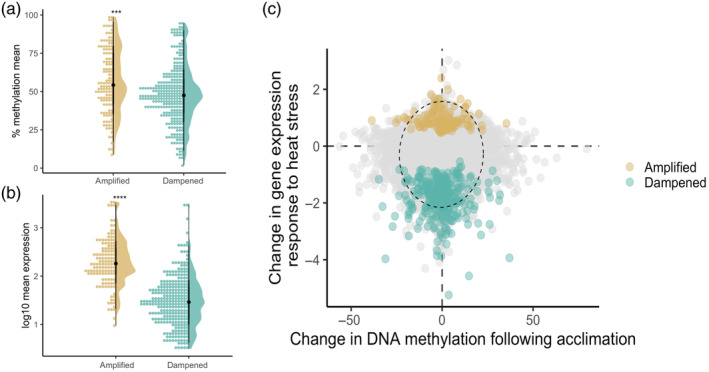
(a) Plot of mean percent methylation of amplified and dampened transcripts. ***p < 0.001. (b) Plot of the log_10_ of mean expression of amplified and dampened transcripts. ****p < 0.0001. (c) Scatterplot of acclimation‐associated gene expression (i.e. Wald statistic) and DNA methylation changes. The oval represents the 99% CI. Legend colors depict genes with significant upregulation and downregulation following acclimation.

We combined gene expression and DNA methylation datasets to examine the potential effects of DNA methylation on gene expression and transcriptional response modulation. There is a small effect but a significant relationship between baseline gene expression and gene‐level baseline DNA methylation (*r* ^2^ = 0.033, *p* < 2e‐16; Figure S4a). This finding aligns with previous studies on gene expression and methylation in metazoans (Dixon & Matz, 2022). We also saw a small negative correlation between DNA methylation percent and the standard error of the log_2_ fold change of heat stress gene expression, a measure that captures variation due to gene expression plasticity and transcriptional noise (*r* ^2^ = 0.025, *p* < 2e‐16; Figure S4b). Together, these results suggest that gene body methylation contributes to preserving baseline gene expression levels and defining the magnitude of gene expression variation. These patterns also explain differences in methylation and expression in genes with transcriptional response modulation. Overall, amplified genes exhibited higher DNA methylation levels compared to dampened genes (Figure 3a; *p* < 0.001) and displayed higher overall expression levels than dampened genes (*p* < 0.0001; Figure 3b).

We were able to investigate the effect of heat stress on DNA methylation independently of the effect of thermal acclimation. Genes that exhibited a shift in methylation in response to heat stress were not the same as the genes within the total set of heat response genes as there was no significant overlap between the expression of the total set of heat stress response genes and differentially methylated genes following heat stress (101 shared genes, chi‐square test, *p* = 0.170). This demonstrates the complexity of the molecular response induced by heat stress processes.

Our primary hypothesis for this study was that transcriptional response modulation was facilitated by shifts in DNA methylation following acclimation within the same genes (Figure 1c). We found no relationship between transcriptional response modulation and differential methylation based on acclimation treatment (Figure 3c). This is supported by the fact that neither amplified nor dampened genes exhibit broad significant changes in DNA methylation as a result of thermal acclimation treatment (Figure S5). However, we did find a larger‐than‐expected overlap between dampened genes and acclimation‐induced differentially methylated genes (14 shared genes, chi‐square test, *p* = 0.009), perhaps suggesting that a subset of differentially methylated genes is also associated with transcriptional response modulation. On the other hand, there was no significant overlap between dampened genes and heat‐stressed induced differentially methylated genes (nine shared genes, chi‐square test, *p* = 0.4470). Moreover, the lack of overlap between amplified genes and differentially methylated genes after thermal acclimation (three shared genes, chi‐square test, *p* = 1) or heat stress (five shared genes, chi‐square test, *p* = 0.3930) provides evidence for a distinct association between specific dampened genes and acclimation‐induced differential methylation. These findings further support the notion that gene body methylation plays a role in maintaining gene expression. However, despite overall correlations between expression, expression plasticity, and methylation, we did not find that short‐term transcriptional modulation is associated with shifts in methylation. Instead, amplified and dampened genes resist methylation changes despite the dynamic methylation patterns observed during acute heat stress and thermal acclimation. While we cannot rule out the possibility that methylation changes may be occurring in large‐effect upstream genes (see Data S1 for details on network analysis, Figure S6), collectively, these results indicate that alterations in gene body methylation are not universally synchronizing the modulation of transcriptional response that we observe.

## 4 DISCUSSION

Acclimation, a form of adaptive phenotypic plasticity, enables organisms to adjust their physiology to survive environmental fluctuations that may be otherwise lethal. Previous studies have shown that acclimation can be associated with shifts in the magnitude of gene expression response to a stressor, in other words, a shift in gene expression plasticity (here referred to as “transcriptional response modulation”) (Bay & Palumbi, 2015). However, the mechanisms preserving the memory of past environmental exposure and mediating gene expression plasticity remain to be resolved in many ecologically relevant species. Here, in the coral *Acropora nana*, we document differential methylation and differences in heat stress‐induced gene expression plasticity between corals acclimated to different temperatures. We find no overall relationship between differential DNA methylation and shifts in gene expression plasticity at the gene level. Interestingly, a small number of genes exhibit both a shift in gene expression plasticity and a shift in methylation level. This observation leads us to propose that DNA methylation shifts are not a general mechanism for controlling short‐term changes in plasticity but may be significant for specific genes. Future studies should focus on alternative mechanisms controlling gene expression plasticity during acclimation.

Gene expression plasticity is critical for homeostasis (Rivera et al., 2021). In a previous study using our same samples, Bay and Palumbi (2015) found transcriptional dampening—the reduction of gene expression plasticity in response to heat stress—in corals more resistant to bleaching due to acclimation at higher temperatures. Our reanalysis replicates these results, finding 446 genes have dampened expression due to the interaction of thermal pre‐conditioning and heat stress. We also identify 206 genes with amplified expression, where the magnitude of gene expression response to heat stress is greater in corals acclimated to higher temperatures. GO term enrichment of amplified genes includes metabolic and transcript processing, mitochondrial components, and cellular membranes. Meanwhile, GO term enrichment of dampened genes is enriched for DNA replication and metabolism. The enrichment of GO terms in amplified and dampened genes suggests that corals acclimated to higher temperatures may optimize gene expression to enhance stress‐response pathways and diminish growth pathway gene expression following thermal acclimation (López‐Maury et al., 2008; Tables S2 and S3).

### 4.1 Timescales of molecular responses to environmental change

In ecological systems, an emerging hypothesis is that DNA methylation mediates adaptive phenotypic plasticity by fluctuating in response to environmental cues and interacting with chromatin‐modifying proteins and transcription complexes resulting in altered gene expression (Eirin‐Lopez & Putnam, 2019; Vogt, 2022). Following thermal acclimation, we identified differential methylation within 416 gene bodies relative to the non‐acclimated samples. This aligns with previous studies finding DNA methylation shifts are associated with various environmental variables, a key aspect of a plasticity mediating mechanism (Crisp et al., 2016; Dimond & Roberts, 2020; Dixon et al., 2018; Eirin‐Lopez & Putnam, 2019; Liew et al., 2018; Metzger & Schulte, 2017; Putnam et al., 2016). In the vertebrate ecological model, three spine stickleback, DNA methylation variation was reported between cohorts of individuals raised in different temperatures, and DNA methylation changed in adults acclimated to different temperatures (Metzger & Schulte, 2017). Recent studies in diverse coral genera reported DNA methylation changes in response to pH, symbiont associations, nutrient stress, and transplantation to novel environments (Dimond & Roberts, 2020; Dixon et al., 2018; Liew et al., 2018; Putnam et al., 2016; Rodriguez‐Casariego et al., 2018). After a 6‐week exposure to acidic conditions, the global methylation percentage doubled in the environmentally sensitive coral species *Pocillopora damicornis* compared to control samples (Putnam et al., 2016). The shift in global DNA methylation brought the methylation percent to a similar level as the more environmentally robust species, *Montipora captitata* (Putnam et al., 2016). In another pH acclimation investigation where *Stylophora pistillata* coral replicates were kept in 4 pH environments for 2 years, changes in DNA methylation due to pH occurred in genes associated with growth and stress response processes (Liew et al., 2018). Together these studies suggest that shifts in methylation can be environmentally induced within the lifetime of an organism. Notably, our experiment was much shorter than previous studies. In this timeframe, we do not observe global shifts in CpG methylation following thermal acclimation (Figure 2b). Instead, we see localized methylation dynamics following the 11‐day thermal acclimation, suggesting that on this timescale, shifts in methylation may be more finely controlled.

Timescales of DNA methylation and gene expression shifts may impact buffering against short‐term fluctuations. In many systems, we know little about how quickly methyl groups can be added or removed from DNA. Our experiment explores two timescales: an 11‐day acclimation and a 24‐h acute stress. Both are quite a bit shorter than many previous investigations. Within other non‐model systems, environmental effects of the methylome have been reported within 48–72 h of environmental stressors during development (Jones & Griffitt, 2022; Strader et al., 2020). Building on findings regarding DNA dynamics after brief stressor exposure, our study revealed that a 24‐h acute thermal stress induced differential expression of 5531 genes and methylation changes in 451 genes. The 11‐day thermal acclimation treatment led to changes in DNA methylation in 416 genes and transcriptional response modulation in 652 genes. DNA methylation can spontaneously occur thus variation can be driven by noise, interfering with the detection of a biological signal between DNA methylation and gene expression (Sanchez & Mackenzie, 2023). However, thermal acclimation and the acute heat stress treatment resulted in both hypermethylation and hypomethylation, indicating the addition and removal of methyl groups presumably by methylation machinery. Previous reports of gene body methylation in marine invertebrates indicate that detectable differences can accrue in a few months. For example, Strader et al. (2020) show that female purple sea urchins conditioned to different abiotic treatments for 4 months produced larvae with differential methylation in 684 genes. In another study, Dixon and colleagues report that highly methylated genes became less methylated, and lowly methylated genes became more methylated in *Acropora millepora* 3 months after transplant (Dixon et al., 2018). Other studies investigating temporal DNA methylation dynamics occur over seasonal timescales where seasonal methylation changes have been reported in gene promoters associated with phenological traits. For example, changes in promoter methylation of transcriptional regulator genes have been identified in the great tit (*Parus major*), which covaries with female reproductive timing (Lindner et al., 2021). However, seasonal stability of gene body methylation across a year was observed in *Arabidopsis halleri* and was associated with stable gene expression (Ito et al., 2019). In the context of previous studies, our study suggests that changes in methylation can occur quite rapidly (within 1 day), suggesting it may contribute to short‐term buffering against rapid environmental fluctuations.

### 4.2 Relationship between gene‐level methylation and gene expression plasticity

We find that methylation is associated with the baseline gene expression level and variation across samples but shifts in methylation do not necessarily affect gene expression plasticity. Previous studies have documented the correlation between gene body methylation and expression across other invertebrate species (Dixon & Matz, 2022; Gatzmann et al., 2018). Additionally, the baseline methylation level is known to be associated with the degree of gene expression plasticity; housekeeping genes have higher methylation levels than environmentally‐inducible genes (Dimond & Roberts, 2016; Dixon et al., 2014; Gatzmann et al., 2018; Sarda et al., 2012). We also see this association between baseline methylation and gene expression plasticity–expression is more variable in genes with lower methylation levels. A potential limitation in our results is in our inability to account for C‐to‐T substitutions between the reference species, *Acropora millepora*, and *Acropora nana*. Still, our results support the same relationships between methylation, baseline gene expression, and expression variation.

Despite the seemingly stable association between methylation and gene expression, we do not find strong evidence that shifts in methylation drive the transcriptional response modulation (Figure 3c); genes with the biggest change in expression magnitude do not show significant changes in DNA methylation. Congruently, the genes with the largest change in DNA methylation following thermal acclimation do not demonstrate a shift in expression magnitude during the thermal challenge. Our findings align with those of Abbott and colleagues, who conducted an independent study on *Acropora millepora*. In their 3‐week thermal acclimation experiment, which involved switching coral fragments from elevated thermal exposure to the control thermal environment at two sampling timepoints, they observed no association between shifts in gene body methylation and either reversible or irreversible shifts in gene expression (Abbott et al., 2024). Furthermore, there were no associations with shifts in the magnitude of gene expression changes (Abbott et al., 2024). Perhaps the general absence of shifts in methylation in genes showing altered plasticity indicates that significant changes in gene body methylation are suppressed in transcriptionally modified coral heat response genes (Muyle et al., 2021; Takuno et al., 2017).

The results also beg the question of whether the acclimation‐induced shifts in methylation have a functional outcome and what that may be. One possibility is that there are trans‐acting effects on gene expression, for example, if acclimation induces methylation in transcription factors (Anastasiadi et al., 2018; Lindner et al., 2021; Moore et al., 2013). Indeed, while we did not find an overall correlation between shifts in methylation and transcriptional response modulation, we did find a small but significant number of genes that exhibited shifts in both methylation and gene expression plasticity, providing candidates for future inquiry. While we find no overall relationship between differential DNA methylation and shifts in gene expression plasticity at the gene level, our gene‐level approach limits our ability to identify the putative role of DNA methylation changes in genes with cascading effects in corals (Gomez‐Campo et al., 2023). Another hypothesis is that DNA methylation shifts could follow changes in expression (Li et al., 2018). This hypothesis is supported by the bimodal DNA methylation in housekeeping and environmentally responsive genes (Dimond & Roberts, 2016; Dixon et al., 2014; Gatzmann et al., 2018; Sarda et al., 2012). Although we do not observe a change in gene expression during the acclimation treatment, it is possible that our sample timing does not capture a change in gene expression during the acclimation period. Another reason why we might not observe a change in the expression of genes with altered DNA methylation following acclimation is that DNA methylation changes are necessary to stabilize gene expression in altered environments (i.e., transcriptional homeostasis) (Li et al., 2018). Since thermal acclimation results from multiple processes that functionally coalesce to shift the baseline temperature at which homeostasis is maintained, it makes sense that acclimation‐induced methylation changes would act to maintain transcriptional homeostasis during subsequent heat stress.

### 4.3 Opposing directions of gene expression plasticity suggest multiple mechanisms

Independent mechanisms operate separately on gene expression and gene expression plasticity across multiple metazoan species, as indicated by the poor correlation between gene expression and plasticity (Xiao et al., 2019). Gene expression is determined by many mechanisms, for example, transcription factor binding, eQTLs, and 3D chromatin organization (López‐Maury et al., 2008). Meta‐analyses of various metazoan species performed by Xiao and colleagues find that cis‐elements and trans‐acting factors promote gene expression plasticity, while epigenetic histone modifications—H3K36me3, H3K79me2 and H4K20me1—inhibit plasticity (Xiao et al., 2019). Here, we identify two modes of transcriptional response modulation: amplified and dampened expression plasticity. There is a relationship between the directionality of gene expression plasticity, gene body methylation, and overall expression (Figure 3b,c); amplified genes have higher overall expression and DNA methylation than dampened genes. The directional changes in expression plasticity, considered with gene body methylation level, may convey that distinct mechanisms are either increasing or decreasing expression plasticity, and the exact mechanism of plasticity mode may be particular to the genetic network (Herman & Sultan, 2011). However, these two modes may not necessarily be acting independently as a small number of genes can largely influence gene expression plasticity (López‐Maury et al., 2008; Schlichting & Smith, 2002).

The relationship between amplified and dampened gene expression plasticity and DNA methylation might be explained by methylation interactions with other mechanisms that cause variation in gene expression. For example, Li and colleagues demonstrate the interaction between DNA methylation and epigenetic histone mark, histone 3 lysine 36 trimethylation (H3K36me3) in Aiptasia (Li et al., 2018). Typically found in gene bodies, this histone mark recruits DNA methyltransferase, which methylates cytosine nucleotides (Li et al., 2018; Weinberg et al., 2019). Li et al. found that DNA methylation was associated with a reduction in transcriptional noise—an expression variation distinct from transcriptional plasticity—where methyl groups potentially inhibit access to cryptic promoters and affect the binding affinity of transcription factors (Héberlé & Bardet, 2019; Li et al., 2018). Methylation‐sensitive transcription factors have been identified across many phyla, indicating that interactions between DNA methylation and trans‐acting factors may be conserved, yet, these remain to be identified in coral species (de Mendoza et al., 2019). Elucidating the relationship between amplified and dampened plasticity and DNA methylation will be an exciting direction for future research.

## 5 CONCLUSION

Prior to our study, gene body methylation was a compelling candidate for establishing a cellular memory of past environmental exposure to influence gene expression plasticity. DNA methylation is a labile chemical mark that can change based on the environment experienced by the organism (Eirin‐Lopez & Putnam, 2019). It is stable yet reversible, and environmentally responsive genes tend to have lower methylation than housekeeping genes (Dixon & Matz, 2022; Gatzmann et al., 2018). Depending on the genomic context, this simple chemical mark is associated with different expression effects. Recent reports indicate that various phyla show no relationship between genome‐wide changes in DNA methylation and change in gene expression plasticity (Bogan & Yi, 2024; Dixon & Matz, 2022; Duncan et al., 2022). Our study, along with the recent paper by Abbott and colleagues (Abbott et al., 2024), suggests that, more generally, cis‐acting differential methylation of gene bodies is not directly responsible for shifts in gene expression plasticity. Our results and other reports of gene body methylation function suggest that the relationship between gene body methylation and gene expression is complex and may depend on gene‐specific cis‐ and trans‐factors and other epigenetics layers. Developing a more thorough understanding of the links between gene expression and epigenetic layers will further our understanding of ecologically important forms of phenotypic plasticity, including potential rates and limits to acclimation in the face of rising temperatures.

## FUNDING INFORMATION

This work was funded by a Sloan Early Career Fellowship and a Packard Fellowship in Science and Engineering to R.B.

## CONFLICT OF INTEREST STATEMENT

The authors have no conflicts of interest related to this publication.

## Supporting information


Data S1:

## Data Availability

Whole‐genome bisulfite sequencing and RNASeq data are available in the NCBI BioProject database under the accession number PRJNA1074434. The metadata tables for gene expression and methylation data, a matrix of gene expression and a matrix of gene level methylation percentage are available of Dryad (doi:10.5061/dryad.76hdr7t3q). All R scripts for this study are available on GitHub (https://github.com/leaguerrero/project‐anana‐thermal‐acclimation).
