# Supplementary material for: Patterns of methylation and transcriptional plasticity during thermal acclimation in a reef‐building coral
Source: Evol Appl. 2024 Jul 17;17(7):e13757. doi: 10.1111/eva.13757 (PMC11254580; doi:10.1111/eva.13757)

Supplemental Methods, Figures and Tables

Supplementary methods:

Accounting for *A.nana* C-to-T substitutions
Congener alignment can complicate methylation calling since C-to-T substitutions may be due to fixed genetic differences between *A. millepora* (reference genome species) and *A. nana* (query species) rather than the unmethylated cytosine conversion to thymine following bisulfite conversion. We used the RNASeq data to estimate how many C-to-T SNPs in the CpG dinucleotide motif exist in *A.nana* coding regions.

We used bcftools mpileup v 1.10.2 followed by bcftools call on sorted RNASeq BAM files data to call multiallelic SNPs between *A.nana* and *A. millepora.* We then used bcftools +fill-tags followed by bcftools convert to create annotated VCF files for downstream filtering. To filter for SNPs with an alternate allele frequency of 1 (i.e. fixed non-reference variants), a read depth of at least 10, variants present in at least 80% of the samples and a minimum genotype quality of 30. To identify C-to-T substitutions, we used bcftools view to identify SNPs where the reference was 'C' and the alternate was 'T'. This filtering we identified 186,786 fixed substitutions within our RNA-Seq data of 23,601 were C-to-T mutations.

In our analysis, we focus on CpG methylation, which means only C-to-T mutations in the CG context would be interpreted as unmethylated. We used samtools faidx to create a FASTA file of the reference dinucleotide motifs for each of the reported positions of the *A. nana* C-to-T SNPs. We found that 5,308 of C-to-T *A. nana* SNPs in the reference CpG motifs. Within *A. millepora* coding regions, there are 1,201,250 CGs. Based on these numbers, a rough estimate of the fraction of CGs that represent fixed differences between species is 0.47%. While we acknowledge the many caveats to this exact estimate (e.g. SNP estimates from RNA-Seq, variable coverage across genes, etc.), this does lead us to believe that a very small fraction of the CpG sites at which we are estimating methylation represent fixed substitutions between the two species.

Differential methylation enrichment in WGCNA expression modules
We conducted a network analysis to test whether genes that were differentially methylated between acclimation treatments were acting as 'hub genes', controlling larger-scale downstream expression changes. We used iterativeWGCNA based on the matrix of normalized gene expression counts to build a gene expression network. We defined 88 well supported modules representing 15,931 genes.

For each module, we conducted two tests. First, we ran an ANOVA testing the effects of heat stress treatment, acclimation treatment, and the interaction with the module eigenvector as the response variable. This tells us which gene expression modules are regulated according to our treatments and identified modules that exhibit the 'amplified' or 'dampened' gene expression response (i.e. those with significant interaction term). Second, we ran a Fisher's exact test to determine whether genes that were differentially methylated during acclimation were overrepresented in a module. Our expectation based on these tests was that if many differentially methylated genes were driving an overall gene expression pattern associated with acclimation response (the "Type I sub-network" from Gomez-Campo et al. 2023), we should see enrichment of methylation genes in modules that represent the gene expression to response to acclimation. We found six modules with enrichment of differentially methylated genes. However, none of these modules had significant acclimation or interaction terms.

We also did a second analysis to determine whether differentially methylated genes represented 'hub genes' – possible regulators of large-scale changes in gene expression ("Type II sub-networks" from Gomez-Campo et al.). We imported the network created with iterativeWGCNA into the R package *igraph*. This package allows us to calculate Kleinburg's hub centrality score for each gene in our network. We then tested whether differentially methylated genes were more likely to be hub genes than other genes in the network. We tested this hypothesis for two sets of genes (nodes): i) all genes in the network and ii) genes in modules with an acclimation effect. Supplementary Figure 6 shows the results for (i) but in both cases differentially methylated genes did not have higher hub scores than the null distribution based on all genes.

Based on these two analyses there is no evidence that differential methylation of hub genes is the mechanism for gene expression plasticity in our experiment (Figure S6). There are multiple reasons that our results likely differ from those in Gomez-Campo et al, the most obvious being that the timescale differs drastically, with the phenotypic plasticity we document occurring on much shorter timescales and resulting in far fewer methylation changes.

In invertebrates, network analysis may not capture biologically meaningful connectedness between gene expression hubs and changes in DNA methylation. Treatment driven differences in DNA methylation may not be detectable because the WGCNA framework assumes co-regulation of genes, and it remains to be determined that DNA methylation is co-regulated in invertebrates.

Supplementary Table 1: Gene Ontology (GO) terms of core heat response genes
Table shows the results from a GO enrichment analysis of the core set of heat response genes using the R package, topGO v. 2.50.0. We compared the list of "core" heat response genes against a background of all transcripts that passed our quality filters. Enriched GO terms were identified with the classic Fisher's test with a p-value < 0.01 and at least 10 transcripts within each category. GO terms are contained within ontology types: Biological Processes (BP), Cellular Components (CC), and Molecular Functions (MF).

Type	GO ID	Term	p-value
BP	GO:0060249	anatomical structure homeostasis	0.00012
BP	GO:0001894	tissue homeostasis	0.00013
BP	GO:0065009	regulation of molecular function	0.00013
BP	GO:0050896	response to stimulus	0.00016
BP	GO:0001503	ossification	0.00018
BP	GO:1901652	response to peptide	0.00018
BP	GO:0009888	tissue development	0.00019
BP	GO:1903037	regulation of leukocyte cell-cell adhesion	0.0003
BP	GO:0045859	regulation of protein kinase activity	0.00033
BP	GO:0001819	positive regulation of cytokine production	0.00035
BP	GO:0007159	leukocyte cell-cell adhesion	0.00038
BP	GO:0065008	regulation of biological quality	0.00049
BP	GO:0051607	defense response to virus	0.00049
BP	GO:0140546	defense response to symbiont	0.00049
BP	GO:0002833	positive regulation of response to biotic stimulus	0.00053
BP	GO:0008219	cell death	0.00062
BP	GO:0006950	response to stress	0.00071
BP	GO:0050863	regulation of T cell activation	0.00084
BP	GO:0050793	regulation of developmental process	0.00084
BP	GO:0043549	regulation of kinase activity	0.00089

BP	GO:0009887	animal organ morphogenesis	0.00101
BP	GO:0048771	tissue remodeling	0.00104
BP	GO:0033993	response to lipid	0.00112
BP	GO:0010941	regulation of cell death	0.0012
BP	GO:0032103	positive regulation of response to external stimulus	0.00128
BP	GO:0032496	response to lipopolysaccharide	0.0013
BP	GO:0032147	activation of protein kinase activity	0.00136
BP	GO:0002764	immune response-regulating signaling pathway	0.00148
BP	GO:0060562	epithelial tube morphogenesis	0.00158
BP	GO:0010557	positive regulation of macromolecule biosynthetic process	0.00172
BP	GO:0009725	response to hormone	0.00172
BP	GO:0001932	regulation of protein phosphorylation	0.00184
BP	GO:0044057	regulation of system process	0.00187
BP	GO:1901617	organic hydroxy compound biosynthetic process	0.00187
BP	GO:0045944	positive regulation of transcription by RNA polymerase II	0.00191
BP	GO:0009607	response to biotic stimulus	0.00191
BP	GO:0048871	multicellular organismal homeostasis	0.00191
BP	GO:1902105	regulation of leukocyte differentiation	0.00199
BP	GO:0045595	regulation of cell differentiation	0.00211
BP	GO:0048513	animal organ development	0.00212
BP	GO:0042221	response to chemical	0.00214
BP	GO:0006915	apoptotic process	0.00219
BP	GO:0002237	response to molecule of bacterial origin	0.0023
BP	GO:0010033	response to organic substance	0.00237
BP	GO:0050790	regulation of catalytic activity	0.00241
BP	GO:0051249	regulation of lymphocyte activation	0.00253
BP	GO:1903706	regulation of hemopoiesis	0.00256
BP	GO:0006066	alcohol metabolic process	0.0026
BP	GO:1901615	organic hydroxy compound metabolic process	0.00263
BP	GO:0048646	anatomical structure formation involved in morphogenesis	0.00266
BP	GO:1903131	mononuclear cell differentiation	0.00267
BP	GO:0048518	positive regulation of biological process	0.00277
BP	GO:0035239	tube morphogenesis	0.00289
BP	GO:0051091	positive regulation of DNA-binding transcription factor activity	0.00296
BP	GO:0043207	response to external biotic stimulus	0.00325
BP	GO:0051707	response to other organism	0.00325
BP	GO:0051247	positive regulation of protein metabolic process	0.00328
BP	GO:1901698	response to nitrogen compound	0.00338
BP	GO:0045893	positive regulation of DNA-templated transcription	0.00339
BP	GO:1903508	positive regulation of nucleic acid-templated transcription	0.00339
BP	GO:1902680	positive regulation of RNA biosynthetic process	0.00346
BP	GO:0006955	immune response	0.00368
BP	GO:0045597	positive regulation of cell differentiation	0.0037
BP	GO:0012501	programmed cell death	0.00372
BP	GO:0010035	response to inorganic substance	0.00375
BP	GO:0002253	activation of immune response	0.00382
BP	GO:0051338	regulation of transferase activity	0.0039
BP	GO:0002694	regulation of leukocyte activation	0.00392
BP	GO:0009891	positive regulation of biosynthetic process	0.00394
BP	GO:0006952	defense response	0.00449
BP	GO:0031328	positive regulation of cellular biosynthetic process	0.00452
BP	GO:0044093	positive regulation of molecular function	0.00452
BP	GO:0043410	positive regulation of MAPK cascade	0.00452
BP	GO:0048522	positive regulation of cellular process	0.00456
BP	GO:0070372	regulation of ERK1 and ERK2 cascade	0.00463
BP	GO:0042981	regulation of apoptotic process	0.00487

BP	GO:0051094	positive regulation of developmental process	0.00494
BP	GO:0044419	biological process involved in interspecies interaction between organisms	0.00497
BP	GO:0042592	homeostatic process	0.0053
BP	GO:0042391	regulation of membrane potential	0.00534
BP	GO:0009617	response to bacterium	0.00555
BP	GO:0070371	ERK1 and ERK2 cascade	0.00558
BP	GO:0051098	regulation of binding	0.00613
BP	GO:0032101	regulation of response to external stimulus	0.00629
BP	GO:0050865	regulation of cell activation	0.00632
BP	GO:0001816	cytokine production	0.00653
BP	GO:0043067	regulation of programmed cell death	0.00713
BP	GO:0001666	response to hypoxia	0.00725
BP	GO:0048856	anatomical structure development	0.00759
BP	GO:0019221	cytokine-mediated signaling pathway	0.0076
BP	GO:0051254	positive regulation of RNA metabolic process	0.00764
BP	GO:0010604	positive regulation of macromolecule metabolic process	0.00765
BP	GO:0002009	morphogenesis of an epithelium	0.00801
BP	GO:0043269	regulation of ion transport	0.00895
BP	GO:0001817	regulation of cytokine production	0.00914
BP	GO:0032502	developmental process	0.00951
BP	GO:0009628	response to abiotic stimulus	0.00979
BP	GO:0036293	response to decreased oxygen levels	0.00996
CC	GO:0031226	intrinsic component of plasma membrane	0.00011
CC	GO:0005615	extracellular space	0.00107
CC	GO:0098802	plasma membrane signaling receptor complex	0.00366
CC	GO:0005581	collagen trimer	0.00386
CC	GO:0005938	cell cortex	0.00396
CC	GO:0098552	side of membrane	0.00865
MF	GO:0005507	copper ion binding	0.0001
MF	GO:0001653	peptide receptor activity	0.00016
MF	GO:0005126	cytokine receptor binding	0.00024
MF	GO:0044389	ubiquitin-like protein ligase binding	0.0004
MF	GO:0031625	ubiquitin protein ligase binding	0.00078
MF	GO:0004497	monooxygenase activity	0.00133
MF	GO:0005102	signaling receptor binding	0.00158
MF	GO:0016705	oxidoreductase activity, acting on paired donors, with incorporation or reduction of molecular oxyge...	0.00246
MF	GO:0016616	oxidoreductase activity, acting on the CH-OH group of donors, NAD or NADP as acceptor	0.00814

Supplementary Table 2: Gene Ontology (GO) terms of Amplified Genes

Table shows the results from a GO enrichment analysis of amplified genes using topGO v. 2.50.0. We compared the list of amplified genes against a background of all transcripts that passed our quality filters. Enriched GO terms were identified with the classic Fisher’s test with a p-value < 0.01 and at least 10 transcripts within each category. GO terms are contained within ontology types: Biological Processes (BP), Cellular Components (CC), and Molecular Functions (MF).

Type	GO ID	Term	p-value
BP	GO:1901566	organonitrogen compound biosynthetic process	0.0002
BP	GO:0009150	purine ribonucleotide metabolic process	0.00023
BP	GO:0009259	ribonucleotide metabolic process	0.00031
BP	GO:0019693	ribose phosphate metabolic process	0.00033
BP	GO:0006163	purine nucleotide metabolic process	0.00036
BP	GO:0042254	ribosome biogenesis	0.00036

BP	GO:0055086	nucleobase-containing small molecule metabolic process	0.00043
BP	GO:1901137	carbohydrate derivative biosynthetic process	0.00068
BP	GO:1901135	carbohydrate derivative metabolic process	0.0007
BP	GO:0072521	purine-containing compound metabolic process	0.00081
BP	GO:0022613	ribonucleoprotein complex biogenesis	0.00086
BP	GO:0090407	organophosphate biosynthetic process	0.00095
BP	GO:0009117	nucleotide metabolic process	0.001
BP	GO:0006753	nucleoside phosphate metabolic process	0.00108
BP	GO:0006397	mRNA processing	0.00151
BP	GO:0006396	RNA processing	0.00234
BP	GO:0032787	monocarboxylic acid metabolic process	0.00277
BP	GO:0044283	small molecule biosynthetic process	0.00614
BP	GO:0016071	mRNA metabolic process	0.00666
BP	GO:0043604	amide biosynthetic process	0.00721
BP	GO:0043603	cellular amide metabolic process	0.00761
BP	GO:0071704	organic substance metabolic process	0.0081
CC	GO:0005743	mitochondrial inner membrane	0.00019
CC	GO:1902494	catalytic complex	0.0002
CC	GO:0005739	mitochondrion	0.00025
CC	GO:1990904	ribonucleoprotein complex	0.00049
CC	GO:0019866	organelle inner membrane	0.00055
CC	GO:0005737	cytoplasm	0.00058
CC	GO:0005740	mitochondrial envelope	0.00069
CC	GO:0031966	mitochondrial membrane	0.0012
CC	GO:0031974	membrane-enclosed lumen	0.00344
CC	GO:0043233	organelle lumen	0.00344
CC	GO:0070013	intracellular organelle lumen	0.00344
CC	GO:0140513	nuclear protein-containing complex	0.00543
CC	GO:0140535	intracellular protein-containing complex	0.00558
MF	GO:0003824	catalytic activity	0.00432

Supplementary Table 3: Gene Ontology (GO) terms of Dampened Genes

Table shows the results from a GO enrichment analysis of dampened genes using topGO v. 2.50.0. We compared the list of dampened genes against a background of all transcripts that passed our quality filters. Enriched GO terms were identified with the classic Fisher's test with a p-value < 0.01 and at least 10 transcripts within each category. GO terms are contained within ontology types: Biological Processes (BP), Cellular Components (CC), and Molecular Functions (MF).

**Type**	**GO ID**	**Term**	**p-value**
BP	GO:0006260	DNA replication	0.00081
BP	GO:0051276	chromosome organization	0.00214
BP	GO:0006259	DNA metabolic process	0.00702
CC	GO:0005694	chromosome	0.0031
MF	GO:0140657	ATP-dependent activity	0.0033
MF	GO:0003677	DNA binding	0.005
MF	GO:0015075	ion transmembrane transporter activity	0.0059
MF	GO:0005488	binding	0.0061
MF	GO:0005215	transporter activity	0.0069
MF	GO:0022836	gated channel activity	0.0084
MF	GO:0015318	inorganic molecular entity transmembrane transporter activity	0.0086
MF	GO:0005216	ion channel activity	0.0088

Supplementary Table 4: Gene Ontology (GO) terms of Differentially Methylated Genes after Heat Stress Assay

Table shows the results from a GO enrichment analysis using topGO v. 2.50.0 of genes that have differential methylation following 24-hour heat stress assay. We compared the list of genes with a methylation percent change of at least 25% and a q-value ≤ 0.05 against a background of all genes included in the methylation dataset that passed our quality filters. Enriched GO terms were identified with the classic Fisher's test with a p-value < 0.01 and at least 10 transcripts within each category. GO terms are contained within ontology types: Biological Processes (BP), Cellular Components (CC), and Molecular Functions (MF). Note that no MF GO terms were enriched in this gene set.

Type	GO ID	Term	p-value
BP	GO:1901564	organonitrogen compound metabolic process	0.0043
CC	GO:0005622	intracellular anatomical structure	0.0018
CC	GO:0043231	intracellular membrane-bounded organelle	0.0032
CC	GO:0043227	membrane-bounded organelle	0.0033
CC	GO:0005737	cytoplasm	0.0057

Supplementary Figure 1.
The Bar plot shows the relative number of reads that align to *Symbiodinium goreaui* or *Durusdinium trenchii* in all samples.

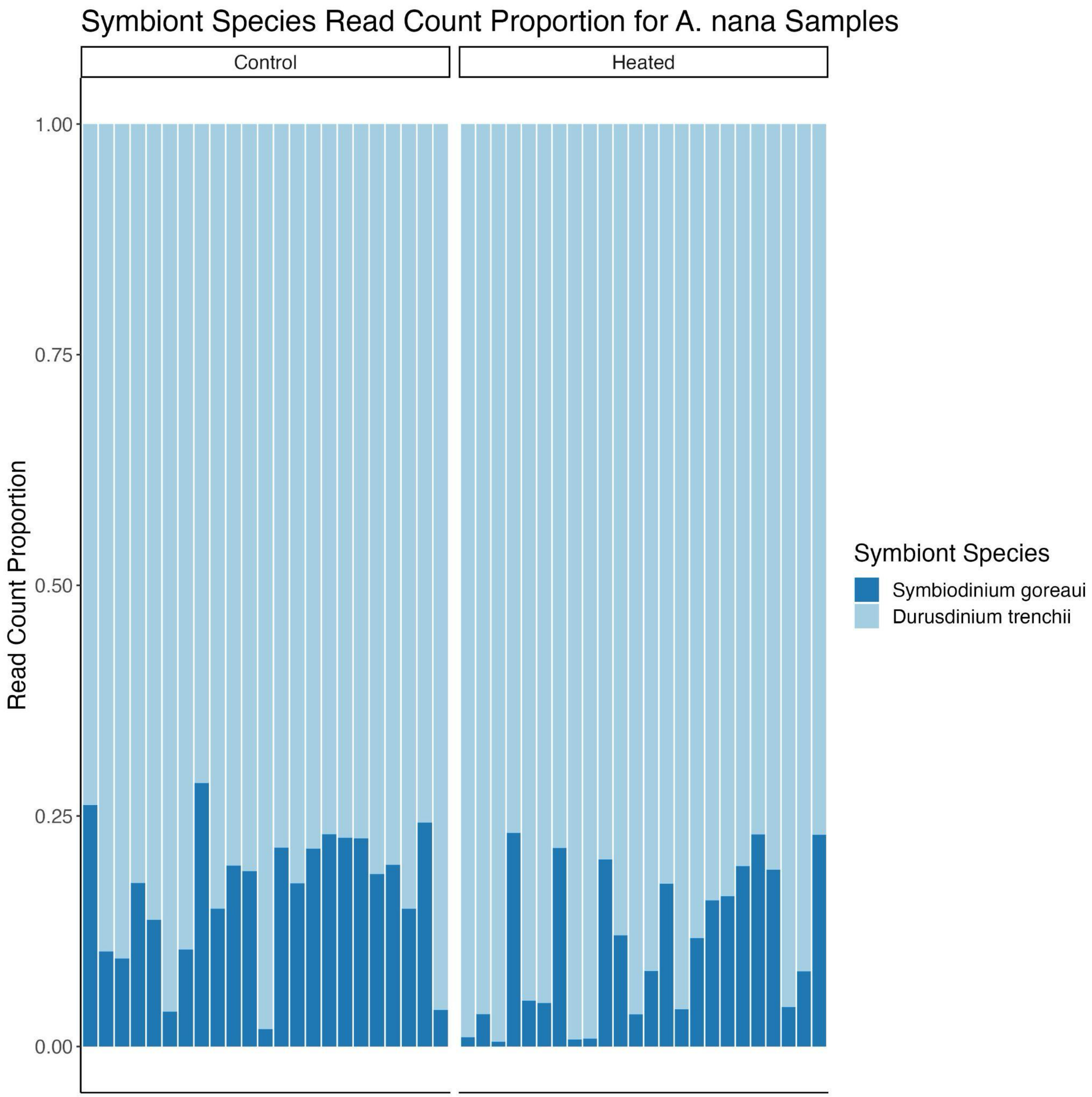

Supplementary Figure 2.
PCoA of DNA methylation before outliers (encircled) were removed. Removed outliers were: 29°C acclimation treatment (Day 11) heat stress sample replicate 1, 29°C acclimation treatment (Day 11) heat stress control replicate 1, 31°C acclimation treatment (Day 11), heat stress treatment replicate 1 and 31°C acclimation treatment (Day 11), heat stress treatment replicate 3.

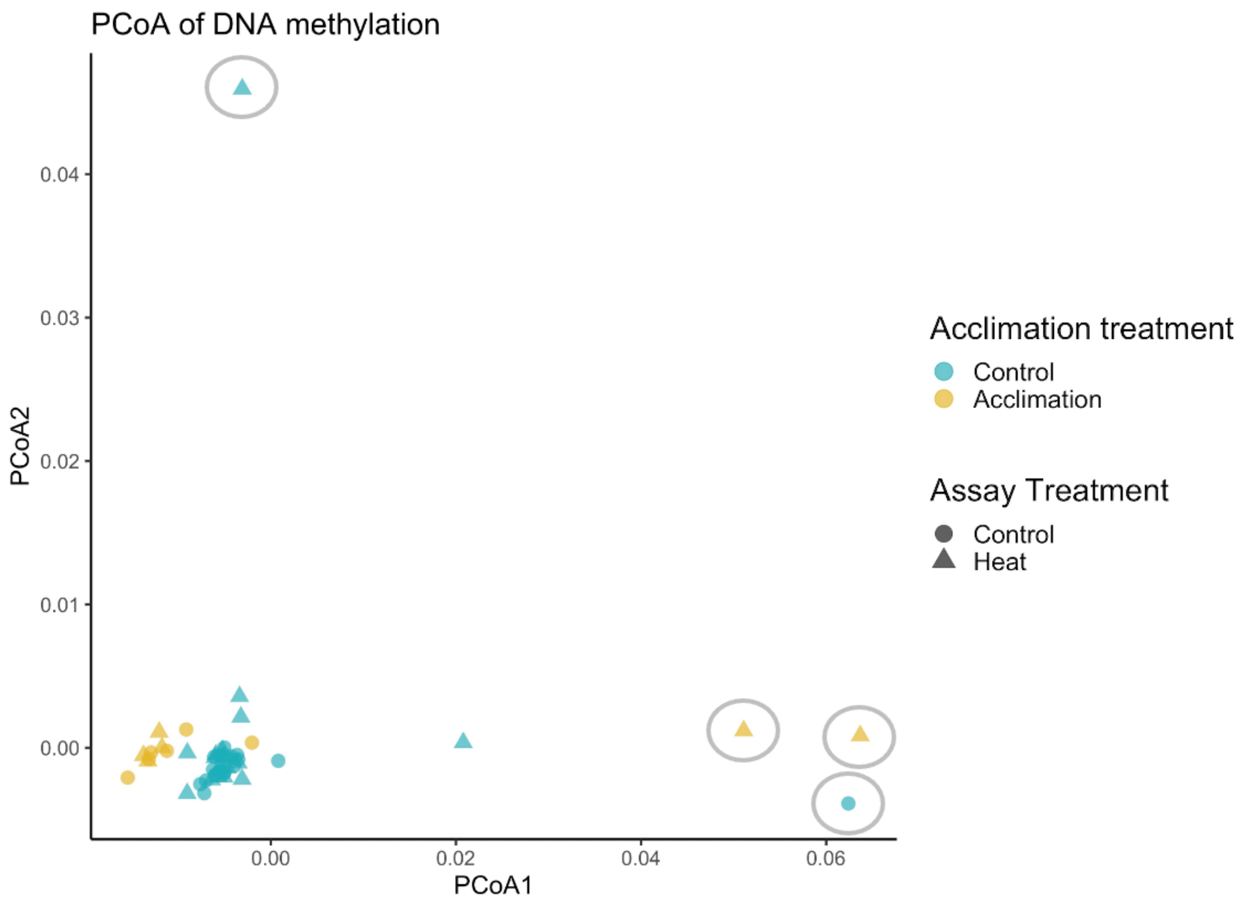

Supplementary Figure 3.

A. Plot showing the bimodal distribution of CpG percent methylation across the genome.
B. Plot showing the distribution of gene-level percent methylation.
C. Distribution of percent methylation within genomic features. The dashed horizontal line represents the median of the feature-specific percent methylation means.

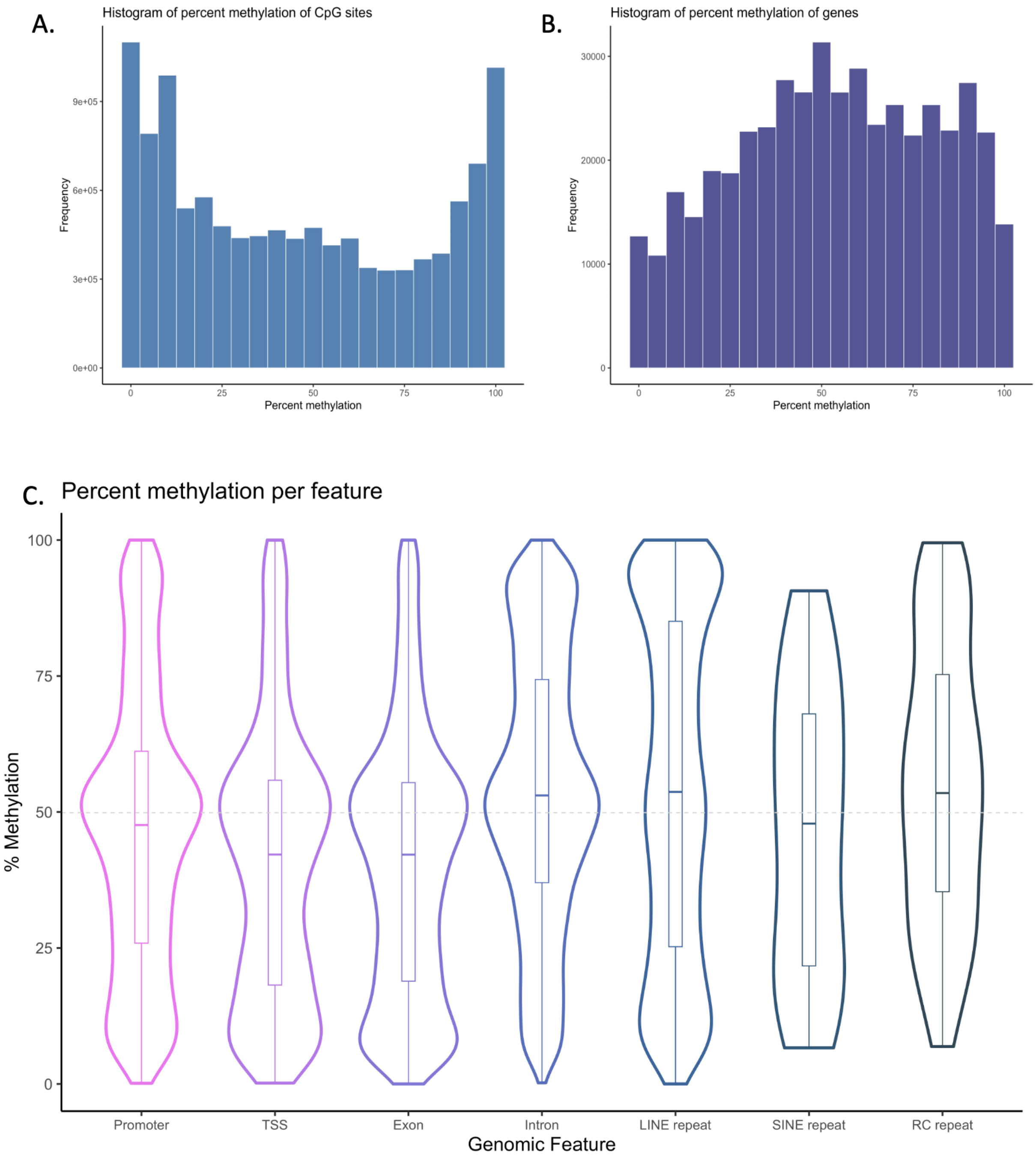

Supplementary Figure 4.

A. Relationship between gene body methylation and baseline expression. On the x-axis is the average percent methylation of genes from all samples. On the y-axis is the $\log_{10}$ transformed expression means of transcripts from all samples.

B. Relationship between gene body methylation and variation in gene expression response to heat stress. On the x-axis is the average percent methylation of genes from all samples. On the y-axis is the standard error of the $\log_2$ fold change between heat stress and control samples.

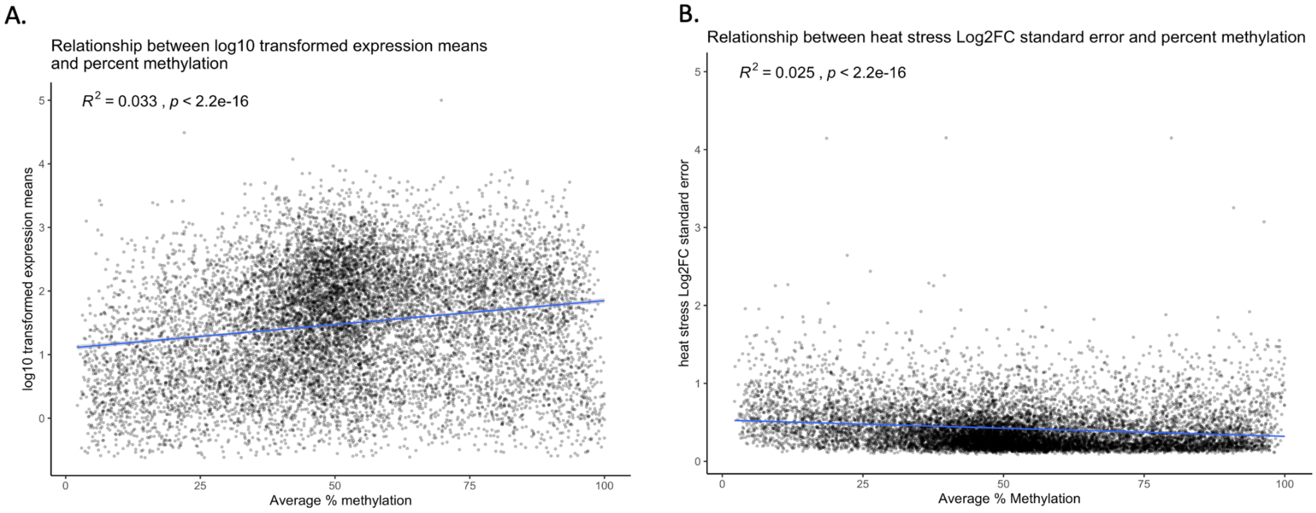

Supplementary Figure 5.
Boxplot of gene body percent methylation of amplified and dampened genes in thermally acclimated samples and the acclimation controls. A t-test was used to compare the means of percent methylation between genes with amplified expression and genes with dampened expression in acclimated samples and control samples.

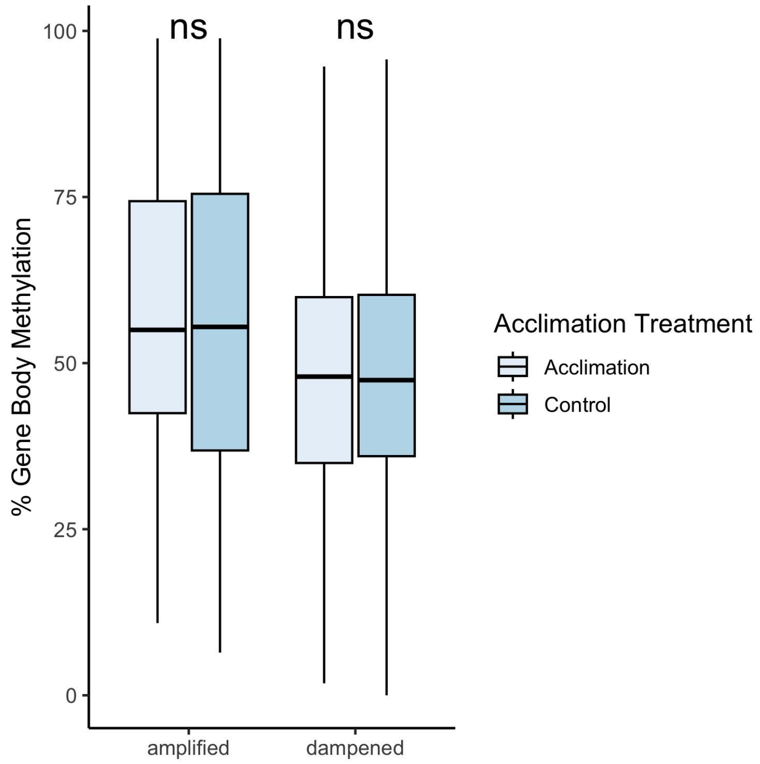

Supplementary Figure 6.

Hub scores for genes in iterativeWGCNA network. Gene categories are amplified (amp), dampened (damp), differentially methylated (meth), and none. Hub scores for 'meth' genes are not significantly different than 'none'.

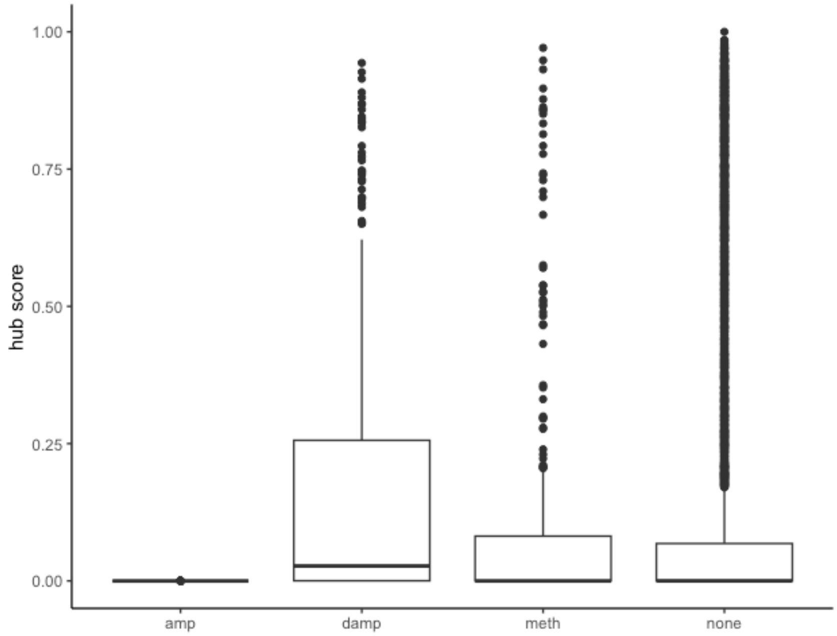

Supplement: Supplementary file 1 — Data S1: [file EVA-17-e13757-s001.pdf]
